# Online analysis of D-glucose and D-mannose aqueous mixtures using Raman spectroscopy: an in silico and experimental approach

**DOI:** 10.1080/21655979.2021.1955550

**Published:** 2021-07-24

**Authors:** Vincent Dumouilla, Claude Gilles Dussap

**Affiliations:** a CNRS, SIGMA Clermont, Institut Pascal, Université Clermont Auvergne, Clermont-Ferrand, France; b Biotechnology and Process Department, Roquettes Frères, Lestrem, France

**Keywords:** Bioprocesses, online raman spectroscopy, carbohydrate’s chemistry, in silico raman analysis

## Abstract

Raman spectroscopy was applied to an aqueous solution containing D-mannose and D-glucose at a fixed dry matter content. The Raman measurement apparatus was adapted online at the industrial scale to monitor a bioprocess including an epimerization reaction. Online Raman spectroscopy and deconvolution techniques were successfully applied to monitor in real time the D-mannose and D-glucose concentrations using the Raman shifts at 960 cm^−1^ and 974 cm^−1^ respectively. The two anomeric forms, α and β of D-mannose in the pyranose conformation were quantified. In silico analysis of vibrational frequencies and Raman intensities of hydrated structure of D-mannose and D-glucose in the pyranose form for α and β anomers were carried out using a two-step procedure. First molecular dynamics was used to generate the theoretical carbohydrates’ structures keeping the experimental dry matter content, then quantum mechanics was used to compute the Raman frequencies and intensities. Computed vibrational frequencies are in satisfactory agreement with the experimental spectra considering a hydration shell approach. Raman intensities are qualitatively in accordance with the experimental data. The interpretation of Raman frequencies and intensities led to acceptable results regarding the current possible structures of D-mannose and D-glucose in aqueous solution. Online Raman spectroscopy coupled with in silico approaches such as quantum mechanics and molecular dynamics methodology is proved to be a valuable tool to quantify the carbohydrates and stereoisomers content in complex aqueous mixtures. This methodology offers a new way to monitor any bioprocesses that encounter aqueous mixtures of D-glucose and D-mannose.

## GRAPHICAL ABSTRACT

**Figure:**
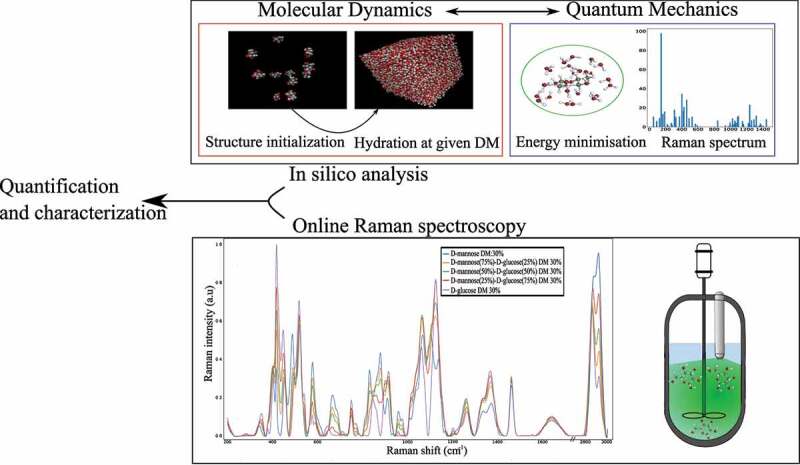

## 1. Introduction

Process analytical technology is a field that encompasses the analysis and control of manufacturing processes while measuring critical process parameters that are related to major quality attributes of a given product. Vibrational spectroscopy, including online Raman spectroscopy, is one of the most commonly nondestructive methods used for process analytical measurements. It is an effective way of assessing critical information regarding the structure of a product which is closely linked to its physicochemical properties. Online Raman spectroscopy is currently used in several production processes in the food industry [1,2] and in the pharmaceutical industry [3,4]. Online Raman spectroscopy has been adapted to monitor a wide range of applications such as D-glucose monitoring [5], cancer diagnosis [6], proteins identification [7], lipid monitoring [8], and determination of the degree of polymerization in complex carbohydrate mixtures [9].

The direct online quantification of each carbohydrate during the epimerization reaction is mandatory to optimize the transformation bioprocess. In this work, online Raman spectroscopy was used in aqueous mixtures of D-mannose and D-glucose at a constant dry matter content to quantify the epimerization reaction of D-glucose to D-mannose. Monosaccharide quantification, including isomers quantification, in the epimerization reaction is performable using two distinct methodologies. The first one, which is widely used, requires the preparation and transportation of a sample from the processing plant to the analytical department to perform a high-performance liquid chromatography analysis. The second methodology proposed in this study consists of using a process analytical tool directly in the bioprocess to measure in real time the physicochemical property of interest. The quantification of carbohydrates in complex mixtures is achieved using several spectroscopic techniques such as Raman Optical activity [10] and NMR spectroscopy [11]. To this extend, Raman spectroscopy was chosen as it is a sturdy tool to study the molecular structure of carbohydrates especially in water mixtures where the Raman behavior of the water molecule is well known and does not overlap the carbohydrates vibrational response. In order to use Raman spectroscopy as a quantification tool, it is mandatory to discriminate the vibrational response of the species in solution relative to their solvent. Consequently, this analysis relies closely on having sturdy insights of the structure of each species. Such structural information might be obtained through the structural analysis of carbohydrates. This is a remarkable task, which is often performed by coupling several analytic techniques such as NMR spectroscopy [11–13], vibrational analysis: Infrared and Raman spectroscopy [14–16], and in some cases neutron diffraction [17,18]. Such techniques are concomitantly used to have an accurate insight of the most probable three-dimensional structure of each species. In parallel to experimental techniques, in silico simulations such as molecular dynamics and quantum calculations are now widely available to study the structure of carbohydrates in aqueous solutions. Density Functional Theory DFT [19,20], has been recognized as a useful theory to predict the vibrational behavior of simple molecules up to carbohydrates and large molecules such as the β -cyclodextrin [21]. However, the DFT results highly depend on the initialization of the procedure.

To this extend, molecular dynamics is a promising approach. Molecular dynamics (MD) methodology relies on applying classical equations of motion to atoms. The molecules are allowed to interact for a certain period of time in a given media where the pressure and the temperature has been set and the dynamical evolution of the system is monitored.

**Figure 1.**
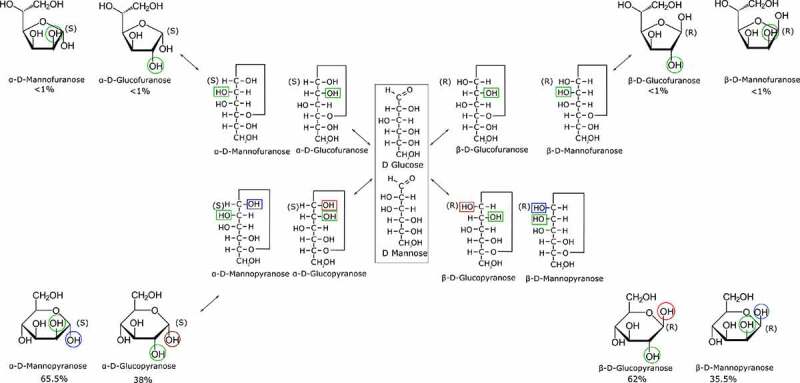
D-glucose and D-mannose structure equilibrium occurring in aqueous solution

The structural difference between D-mannose and D-glucose lies in the chirality of their hydroxyl groups on the C2 carbon (Figure 1). The structural complexity is enhanced when considering the several chemical conformations a monosaccharide may adopt in an aqueous solution. The linear form of the D-glucose and D-mannose molecules is replaced by the pyranose or furanose rings that might exhibit the α or β specification, which corresponds to the specific chirality of the C1 carbon. This phenomenon is called the anomeric equilibrium [22]. In addition, the pyranose form can adopt the 4C1 and 1C4 conformations considering that DFT studies suggest that both forms are probable in aqueous solution [23]. Previous vibrational studies [24,25] showed that Raman spectroscopy allows to assign and characterize the glycosidic bond of the anomeric form of D-glucose. While the emphasis has always remained on the D-glucose molecules, few data are available regarding the behavior of D-mannose monomer in aqueous media.

The present study concerns a structural analysis of a ternary mixture of D-mannose, D-glucose and water. It is performed through experimental online Raman spectroscopy and in silico methodology coupling quantum and molecular dynamics calculation. First, online Raman spectroscopy is applied to monitor D-mannose and D-glucose concentrations in ternary aqueous solutions. Then, an in-silico analysis is developed by analyzing the computed frequencies and Raman intensities, showing that the theoretical calculations reasonably fit with the experimental measurements, opening the door to a large application of Raman spectroscopy for online characterization of carbohydrates biotransformation and bioprocesses.

## 2. Materials and methods

### 2.1 Material

All monosaccharides: D-glucose and D-mannose were obtained from Sigma Aldrich. Solutions were gently heated and continuously stirred in a batch reactor until complete dissolution. All solutions were filtered using Millipore^TM^ Millex syringe PTFE filters (pore size 0.45 μm). All mixtures have a 30% DM (dry matter) concentration.

### 2.2. Raman spectroscopy apparatus

Raman spectra of carbohydrates solutions were recorded using a Tornado HyperFlux^TM^ PRO spectrometer equipped with a 785 nm laser probe in an industrial reactor. The spectrometer resolution is 2 cm^−1^ and the laser power of 480 mW. Due to industrial limitations, the exposure time was set to 45 seconds and the height of the Raman probe was carefully optimized to obtain the maximum intensity for the specific collection geometry. An average of five scans was accumulated to obtain the spectra in the 100–3000 cm^−1^ spectral range.

### 2.3. Signal processing and deconvolution methodology

Raman spectra were treated using a penalized least-square procedure [26]. The fitting is performed by fitting the deconvoluted intensity $I_{Raman,deconvoluted}$, calculated using the sum of the intensity given by the several Gaussian or Lorentzian profiles, to the experimental Raman intensity $I_{Raman,experimental}$.

$$I_{deconvoluted}\left(Gaussian\right)=\underset{n}{\sum }\underset{i}{\sum }A_{n}exp\left[-\frac{1}{2}{\left(\frac{x_{n}-v_{i}}{\sigma_{n}}\right)}^{2}\right]\;\left(1\right)$$

$$I_{deconvoluted}\left(Lorentzian\right)=\underset{n}{\sum }\underset{i}{\sum }A_{n}\frac{1}{1+4{\left(\frac{x_{n}-v_{i}}{\sigma_{n}}\right)}^{2}}\;\left(2\right)$$

$A_{n}$ is the amplitude of the n profile. The $x_{n}$is the extracted Raman frequency at the apex of the profile and $\sigma_{n}$ is the standard deviation that corresponds to the bandwidth. Then, the parameters are fitted by minimizing the difference between the fitted intensity and the experimental intensity. The deconvolution methodology is performed with any programming language using the following equations related to the Gaussian and Lorentzian profiles.

### 2.4. DFT computational details

All Raman spectra were calculated using the 2020 version of Turbomole software provided by Biovia industry [27] using a BP86-TZVP-DFT-DISP3 procedure. The current version of Turbomole quantum package allows the calculation of more detailed energy hypersurfaces, especially for hydrogen bonds (BP86-TZVP-DFT-DISP3) since Grimme dispersion corrections for nonbonded interactions were implemented recently [28]. Raman intensities were computed using the Raman backscattering cross-section (Orth unpolarized), which best represents the experimental geometry with the following relation:

$$\frac{d\sigma }{d\Omega }=\frac{h}{4\pi_{0}^{2}c^{4}}\frac{{\left(\omega -\omega_{v}\right)}^{4}g_{v}}{2\omega_{v}}\left(\frac{45\alpha^{\prime 2}\left(\omega \right)+7\gamma^{\prime 2}\left(\omega \right)}{45}\right)\;\left(3\right)$$

**Figure 2.**
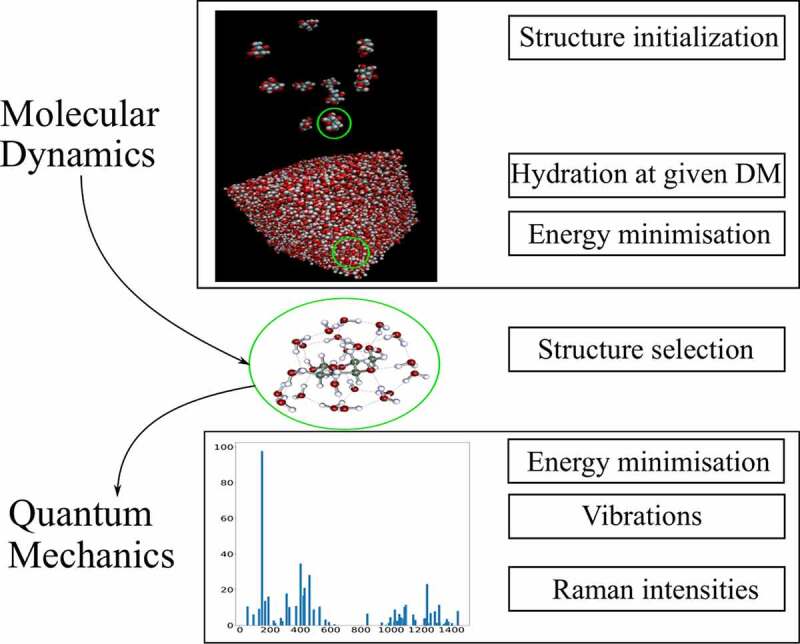
Schematic representation of the structure algorithm used to select the final structure

$\alpha^{\prime 2}\left(\omega \right)$ and $\gamma^{\prime 2}\left(\omega \right)$ denote the isotropic part and the anisotropy of the differentiated polarizability tensor, respectively. The previous relation includes the vibrational frequency $\omega_{v}$ and the degeneracy $g_{v}$ of the vibration, c is the speed of light and $\varepsilon_{0}$stands for the dielectric constant of vacuum. The Raman cross-sections were normalized using the same procedure as for the experimental spectrum. All hydrated structures were obtained using a well-defined methodology (Figure 2) that is validated throughout this study: several molecules of the same monosaccharides were hydrated using the molecular dynamics Gromacs software [29] to the required number of molecules to reach 30% DM. Then, several energy minimizations: NVT (Number of particles, Volume, Temperature) and NPT (Number of particles, Pressure, Temperature) were carried out to set the simulated system to the experimental conditions at 1 bar and 300 K to assess the most likely associations between molecules in the solution through molecular dynamics. Then the monosaccharides structures and their corresponding hydration shells were extracted from the resulting structures, keeping the 30% DM ratio before they were imported in the Turbomole package to achieve a finer DFT energy optimization and vibrational calculation. The calculated Raman intensities regarding each simulated structure were weighted by the anomeric concentration of each species in aqueous solution given by NMR measurements [12].

## 3. Results

### 3.1 Experimental raman spectra of aqueous mixtures of D-glycose and D-mannose

**Figure 3.**
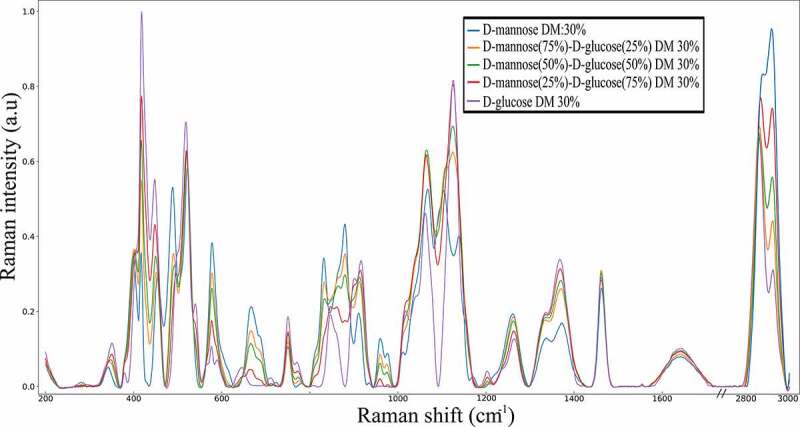
Raman spectra of pure D-glucose D-mannose and the corresponding mixtures of 25%-75%, 50%-50%, 75%-25% ratio of D-mannose and D-glucose in aqueous solution. Every spectrum has a fixed 30% DM

The results of experimental Raman spectra of pure D-glucose and pure D-mannose in aqueous solutions and the mixtures at 25−75%, 50–50%, 75–25% of D-mannose/D-glucose at 30% DM are shown in Figure 3 and Table 1. The results are consistent with the Raman spectra of referenced carbohydrates found in the literature [14,25].

**Table 1.** Raman frequencies for aqueous solution of selected monosaccharides D-glucose, D-mannose and the 50% D-mannose and D-glucose mixture at 30 DM. s-strong, m – medium, w – weak, ν stretching, δ bending, ώ wagging

Spectral region	D-glucose	D-mannose	50% mixture	Assignment
The OH stretching	3400(s)	3400(s)	3400(s)	ν(OH)
The CH2 stretching	2945(s),2898(s), 2721(s)	2942(s), 2909(s),2737(w)	2942(s),2902(s),2729(w)	ν_as_(CH_2_), ν _ss_(CH_2_), ν(CH)
The HOH bending	1645(m)	1645(m)	1645(m)	δ (OH)
The CH2 and CIH deformations	1462(s)	1462(w),	1462(w)	δ (CH_2_),
	1367(s)	1368(w)	1371(w)	δ (CH_2_),
	1336(m)	1336(m)	1336(m)	δ (CH)
	1264(m)	1264(w)	1262(w)	ώ(CH_2_)
The fingerprint	1126(m)	1138(m)	1123(m)	δ (COH)
	-	1103(m)	1105(m)	δ (COH)
	1061(m)	1063(m)	1067(m)	ώ(CH_2_)
	1017(s)	1010(w)	-	δ (COH)
	-	960(m)α,974(m)β	960(m)α,974(m)β	ν(CO)
	899(m),910(m)	910(w)	910(m)	δ (COH)
	-	878(w)	878(w)	δ (COH)
The anomeric	846(m)	831(m)	833(m)	δ (COH)
	774(w)	-	774(w)	δ (COH)
	749(w)	749(w)	749(w)	δ (CCH)
	709(w)	681(m)	681(w)	ν (CCH)
	644(w)	665(m)	665(w)	δ (CH)
The low wavenumber	590(w),577(w)	577(w)	577(w)	-
	540(m)	-	-	-
	518(m)	520(m)	522(m)	-
	497(m)	488(m)	491(m)	-
	447(m)	454(w)	449(w)	δ (CCO)
	417(s)	417(w)	402(m)	δ (CCO)
	407(w)	398(w)	-	δ (CCC)
	350(w)	341(w)	347(w)	δ (CCC)

Vibrational spectra of carbohydrates have been categorized in several spectral ranges, each of them being related to a particular set and movements of atoms.

The highest wavenumber region ~3000–3800 cm^−1^ exhibit vibrational frequencies of the stretching motions (ν) of the hydroxyl groups of water and monosaccharides. It must be pointed out that the intensity of the water frequencies completely overcome the intensity of the hydroxyl groups of the carbohydrates in solution limiting the investigation of the hydration phenomena.

The intensity shifts in the 2800–3000 cm^−1^ region correspond to the stretching vibrations (ν) of the CH_2_ groups of the monosaccharides (Table 1).

The CH_2_ and COH bending vibrations (δ) are observed in the 1200 to 1500 cm^−1^ region [14]. This region includes the vibrations related to the exocyclic conformation of the CH_2_OH group, leading to observe that D-mannose and D-glucose exhibit similar vibrational behavior in this region (Table 1, Figure 3).

The fingerprint region ~950–1200 cm^−1^ exhibits characteristic bands representative of the rings’ conformation and the relative orientation of several substituents [14]. This region concerns the deformation vibration (δ) of C-OH, C-CH, and O-CH groups. The vibrational response in the interval between 600 and 950 cm^−1^ allows the specification of the β and α anomers for D-mannose, the characterization of pyranose and furanose rings (Table 1). It is found that the ratio of the intensities of the 960 and 974 cm^−1^ peaks corresponds to the anomeric proportions of α and β forms (32.2% and 67.8% respectively) so that the following relation holds for estimating the anomeric proportions of D-mannose in aqueous solutions:

$$\alpha D-mannopyranose\left(\%\right)=\frac{I_{960}}{I_{960}+I_{974}}$$

$$\beta D-mannopyranose\left(\%\right)=\frac{I_{974}}{I_{960}+I_{974}}$$

It is an important result as it enable to quantify the D-mannose and D-glucose content in any bioprocesses at the industrial scale. Moreover, this region has been previously used to discriminate the two anomeric forms for the solid D-glucose [25]. The wavenumbers ranging from 350 to 600 cm ^−1^ contain vibrational patterns of the bending motions (δ) of the pyranose cycle including the anomeric oxygen motions.

### 3.2 In silico Raman analysis of D-mannose and D-glucose

The calculated frequencies and Raman intensities for both α and β anomers for D-glucose and D-mannose in the pyranose form are shown in Figure 5.

The simulated hydroxyl stretching motions (ν) in the range 3000–3800 cm^−1^ are specific to water and monosaccharides hydroxyl groups. In each final structure, bond lengths of the hydroxyl groups were elongated from 0.972 Å to approximatively 1.01 Å for each hydroxyl group of water and carbohydrates molecules. Each hydrogen bond length between water–water molecules and monosaccharides-water molecules measured approximately 1.85 Å. The CH, CO, CC bond lengths measured approximately 1.11 Å,1.45 Å,1.52 Å respectively and the angle of the CH_2_ exocyclic measured 103.11° which matches with previous DFT simulations [30].

The fingerprint region from 1100 to 1400 cm^−1^ encountered several modifications: the wagging CH_2_ motions (ώ) observed in both D-glucose and D-mannose spectra at 1365 cm^−1^ and 1336 cm^−1^ (Figure 3, Table 1) are well represented by the simulated structure (Figure 5).

There are 3 n – 6 frequencies for each n atom leading to a total of 231 total frequencies for each structure. Consequently, the analysis has been focused on the anomeric region where the most changes appear at 900–1000 cm^−1^.

The simulated vibrational frequencies for α D-mannose pyranose were located at 956, 971,984, and 993 cm^−1^ and for β D-mannose pyranose at 941, 950, 965, 968, 978, 988, and 991 cm^−1^ respectively. Those frequencies are in accordance with the experimental measurements assessing the 974 cm^−1^ and 960 cm^−1^ experimental shifts to the D-mannose anomeric forms.

For D-glucose, vibrational simulation led to only two frequencies with low intensities in the 941–992 cm^−1^ region (Figure 5) at 969 cm^−1^ and 986 cm^−1^ for the β D-glucose pyranose and α D-glucose pyranose, respectively.

## 4. Discussion

Despite their relatively common structures, monosaccharides exhibit several structural conformations in aqueous solutions which are often encountered in several bioprocesses. The furanose and pyranose rings are tautomer structures in addition to the linear form. Each ring conformation exists in the α ↔ β forms. This is the anomeric equilibrium which concerns a difference in the orientation of the C1 hydroxyl group (Figure 1). The ratio of each structure and their anomeric ratios are different for each monosaccharide. The anomeric ratio for a wide range of monosaccharides in aqueous media has been successfully quantified with NMR spectroscopy [12]. D-Glucose and D-mannose exist in an equilibrium mixture of α and β anomers with the domination of the pyranose form as the furanose form concentration is negligible. In aqueous solution the D-mannose molecule is at 65.5% in the α pyranose form and 34.5% in the β pyranose form while 38% of the D-glucose is in the α pyranose form and 62% is in the β pyranose form in aqueous solution.

**Figure 4.**
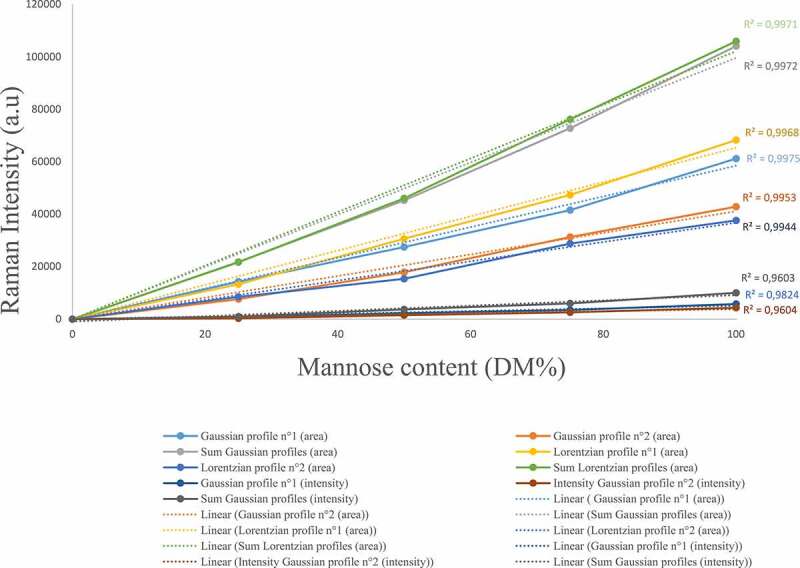
Graphical representation of the relationship between the raw surface area obtained using a deconvolution methodology with Gaussian and Lorentzian profiles and the intensity of Raman apex and the D-mannose dry matter in aqueous solution

The Raman spectra obtained for the D-mannose/D-glucose mixtures of different proportions (25–75%, 50−50%, 75−25%) at 30% DM (Figure 3) exhibits differences regarding the carbohydrate’s concentrations in two specific regions. The 631–710 cm^−1^ and the 941–992 cm^−1^ regions are related to the anomeric equilibrium of D-mannose. In addition, D-glucose does not exhibit any Raman intensity in this region allowing the quantification of D-mannose concentration. To quantify the amount of D-mannose, a gaussian fitting was performed in the 941–992 cm^−1^ spectral region using the deconvolution methodology. Two profiles were sufficient to ensure a satisfactory fitting of the area and have led to characterize the anomeric equilibrium of D-mannose. The carbohydrates content determination was carried out using two distinct methodologies: i) the comparison between the total area under the curve for D-mannose using Lorentzian and Gaussian profiles and, ii) the comparison of the intensity of the two gaussian profiles. The area under the curve methodology yields more accurate results, leading to a linear trend between the D-mannose content and the area under the curve of the two gaussian profile in the (941–992) cm^−1^ spectral range (Figure 4). This behavior is linked to the relationship between the raw Raman intensity and the concentration of the species in solution as stated by previous theoretical work [31]. Interestingly, the ratio of the intensities of the observed Raman peaks at 960 cm^−1^ and 974 cm^−1^ is not dependent on the D-mannose concentration. Since those frequencies are not observed in pure D-glucose solutions, they are attributed only to D-mannose.

None of the observed Raman peaks were suitable to perform a similar analysis to obtain the anomeric proportions for the D-glucose solutions. Several band assignment for α and β D-glucose anomers exits but they are based on the crystallin D-glucose [32] at 1338/1320 cm^−1^, 1224/1278 cm^−1,^ and 1109/1162 cm^−1^ for α and β anomers regarding the D-glucose molecule. Those current markers could not be applied to pure D-glucose aqueous solutions due to Raman bands overlapping and complex water D-glucose interactions. In a previous work [14], 850 cm^−1^ and 918 cm^−1^ Raman frequencies observed for D-glucose aqueous solution were assigned to the α and β pyranose forms respectively. The D-mannose experimental spectrum exhibits a frequency at 850 cm^−1^ and at 918 cm ^−1^ indicating that the previous band assignment might not strictly related to the D-glucose molecule but is a good marker when analyzing pure D-glucose solution. It should be emphasized that Raman spectroscopy might be limited when analyzing epimers with complex structure due to the large number of peaks and the complexity of the Raman signals. This is the case for polysaccharides such as cyclodextrins and disaccharides [14]. To this extend, Raman spectroscopy should be used simultaneously with Raman optical activity and with NMR measurements when the vibrational responses are not sufficient to perform a sturdy analysis.

The following discussion section aims at deriving a predictive methodology based on an in-silico approach for interpreting complex experimental data issued from Raman spectra of aqueous solutions of carbohydrates. The goal here is to determine computed vibrational frequencies and Raman intensities for developing valid descriptors to assess monosaccharides concentrations in aqueous solutions. Since in silico methods such as molecular dynamics and quantum computations are a common way of analyzing molecule behavior in hydrated media, the goal is to determine the regions of the spectra that are specific to different solutes. As vibrational analysis relies on the estimation of the Hessian matrix which is computed using the derivatives of the energy matrixes, the sturdiness of the energy calculation is mandatory.

Recent advances in parallel computing allow DFT tools to compute vibrational frequencies and Raman intensities of large systems. DFT is a quantum mechanics methodology that computes the ground state energy of systems with exchange-correlation functionals incorporated in the Hamiltonian part of the corresponding Schrodinger wave equation, this methodology is used to compute several molecular properties such as vibrational frequencies [33] and the corresponding IR [34] and Raman intensities [35], NMR shielding [36], vibrational circular dichroism and several excited states properties. DFT studies applied to carbohydrates solutions have been carried out to assess the optical rotation of D-glucose [37], and vibrational calculations for the D-glucose molecule. This has led to satisfying results [19,38] even for investigating the anomeric equilibrium for D-glucose [15,39]. However, it must be outlined that DFT calculations require an initial estimation of the molecular structure to give reasonable results.

For the present study, Turbomole data package was first used for creating hydration shells. The results showed that when convergence was obtained the energy minimization algorithm used by Turbomole package was sufficient to eliminate imaginary vibrational frequencies. This is a first-order validation for preventing the simulation of unstable structures. However, due to the huge computational costs, DFT minimization algorithm has been found to be only accurate on small systems [40] with a limited number of water molecules hydrating the carbohydrates. Moreover, it has been observed that convergence has been difficult to obtain when initializing too far from realistic structures, likely to exist in real solutions. In other words, DFT minimization techniques converge toward a local minimum that remains close to the initialization structure.

On the other hand, the molecular dynamics software Gromacs [29] tool can be used directly to derive the most probable structure on which to perform vibrational analysis providing an alternative for initializing the desired structure. Several Molecular dynamics study have been performed regarding carbohydrates in aqueous media [41–43] even for the D-glucose molecule [41] and gives reasonable insights on noncovalent driving force, hydrogen-bonding numbers, free energy of molecule movement, hydrophobic and hydrophilic surface area of the molecules with other components. The study of conformations is a major concern in carbohydrates chemistry as it is one of the main differences between monosaccharides.

With this direct approach, it has been observed that optimized structures obtained by this way happened to have unwanted imaginary frequencies or in unhabitual vibrations range in the 1700–2700 cm^−1^ region that indicates an incorrect or unlikely conformation.

The method developed in this study consists of associating the two former methods taking advantage of both. First, the molecular dynamic tools are used to predict reasonable hydrated structures before a DFT algorithm for finalizing energy minimization and vibrational calculations. This strategy has allowed to conserve the initial hydrated shells without introducing imaginary frequencies. The final methodology consists of reaching the appropriate concentration (30% DM) with forcefield GLYCAM06 [44]. A cascade of several molecular dynamic’s simulations including first-order energy minimizations was carried out. NVT (Number of particles, Volume, Temperature) and NPT (Number of particles, Pressure, Temperature) procedures led to progressively reach the experimental temperature and pressure conditions (1 bar and 300 K). Then the monosaccharide structure with its corresponding hydration shells was extracted while keeping the 30% DM. The structures were imported in the Turbomole package before fine energy minimization with the DFT BP86 DISP-3 theory level. The obtained simulated structures for D-mannose and D-glucose were composed of 23 water molecules surrounding one molecule of carbohydrate. This configuration did not take into account the interactions between carbohydrates molecules considering they are negligible whenever the solute remains solubilized.

The simulated frequencies of OH group measuring approximately 1 Å match the experimental Raman frequencies of liquid water and dissolved monosaccharides at 3400 cm^−1^. The hydrogen bond distances and OH groups bond lengths were in accordance with neutron diffraction measurements obtained in aqueous solutions of monosaccharides where hydrogen bond lengths were in the 1.75 − 1.90 Å range [18]. This indicates that DFT methodology takes into account the hydration phenomena by elongating the hydroxyl group from 0.971 Å to1 Å and by setting a hydration shell of water molecules at a distance of approximately 1.90 Å of the carbohydrate.

**Figure 5.**
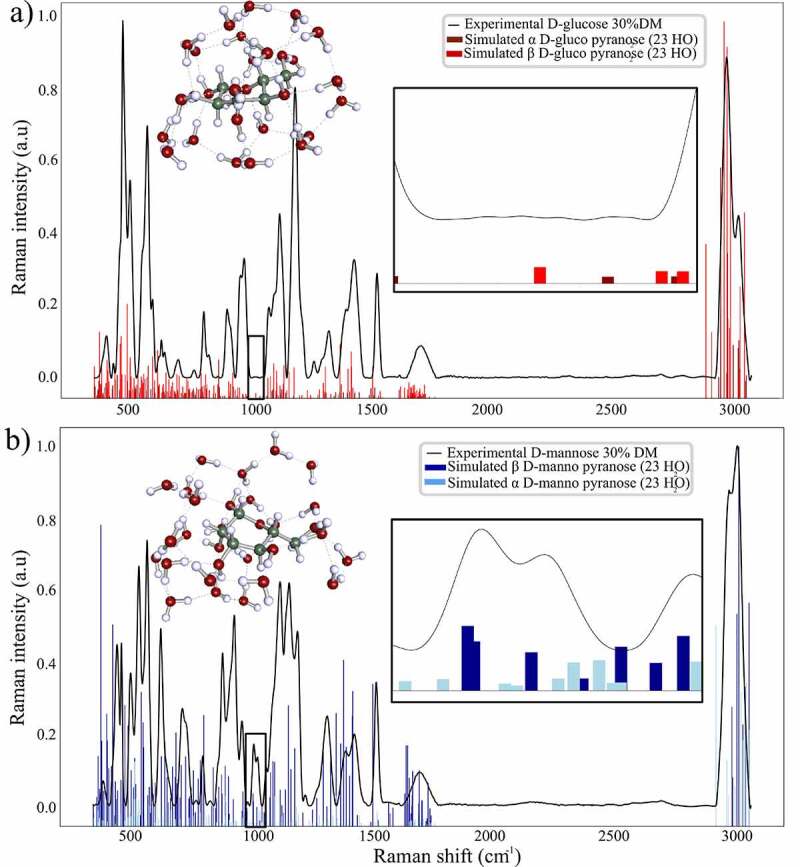
Simulated vibrational frequencies and Raman intensities of α and β pyranose form of D-glucose and D-mannose resulting from molecular dynamics and quantum mechanics energy minimization algorithm expressed versus the corresponding experimental Raman spectrum of the pure compound in aqueous solution at 30%DM

In addition, the deformation vibration (δ) of CH and CH_2_ groups at ~1460 cm^−1^ are correctly modelized for each species (Figure 5), indicating that the geometry or the CHO hydroxyl groups are accurately modelized even when an aqueous environment of water molecules interacts with the monosaccharide.

The calculated Raman frequencies for the simulated D-mannose structures are in accordance with the experimental measurements assessing the 974 cm^−1^ and 960 cm^−1^ experimental shifts to the D-mannose anomeric forms. Moreover, the normal modes of the computed frequencies previously listed for both D-mannose structures clearly involve the C1 anomeric group reinforcing the previous experimental assignment.

The calculated Raman frequencies for the simulated D-glucose structures led to only two frequencies with low intensities in the 941–992 cm^−1^ region (Figure 5) for the β D-glucose pyranose and α D-glucose pyranose. Additionally, the normal modes corresponding to both vibrations involve complex water carbohydrates motions in the methyl region. The remarkably low number of frequencies and the non-anomeric related normal modes for the calculated frequencies and Raman intensities of the D-glucose pyranose structures indicate that the optimized structure for D-glucose are reasonable descriptors of the real structure of D-glucose in an aqueous solution.

While vibrational analysis strictly does not allow to distinguish anomeric forms, the calculated intensity ratio of the sum of the calculated Raman intensities for each anomeric form of D-mannose leads to 61.1% and 38.9% respectively. This is in close agreement with the experimental values confirming the way to quantify the anomeric proportion for D-mannose anomers in aqueous solution.

The developed in silico methodology is not yet accurate enough to claim that the anomeric concentration can be found by combining DFT and molecular mechanic dynamics calculation. But it must be outlined that this methodology gives reasonable insights in the vibrational frequencies and Raman intensities of aqueous mixtures of monosaccharides.

## 5. Conclusion and perspectives

Online Raman spectroscopy was successfully used to assess D-glucose and D-mannose concentrations in aqueous solution. This methodology also assesses the ratios of D-mannose anomers. An approach associating molecular dynamics and quantum mechanics was developed to determine and predict the vibrational frequencies and Raman intensities of monosaccharides in aqueous solutions. The vibrational analysis and interpretation of Raman intensities peaks give satisfactory results regarding the current possible structures of D-mannose and D-glucose in aqueous solution. This new methodology appears to be a valuable tool to characterize aqueous solutions of monosaccharides coupling online Raman spectroscopy and in silico approaches in bioprocesses.
